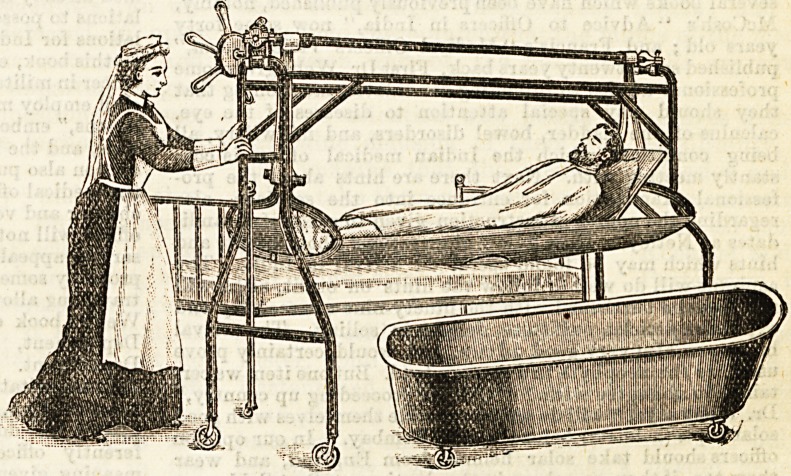# New Drugs, Appliances, and Things Medical

**Published:** 1891-06-13

**Authors:** 


### NEW DRUGS, APPLIANCES, AND THINGS MEDICAL.

[All preparations, appliances, novelties, etc., of which a notice is desired, should be sent for The Editor, to care of The Manager, 140, Strand, London, W.C.]

### A NEW BEDLIFT.

(Jack and Smith's, 58, Seymour Street, Euston Square.)

We have recently inspected a new form of bedlift at Messrs. Jack and Smith's manufactory at the above address

The lift consists of a tubular iron frame on four wheels centreing and passing over the head and foot of the bed. This part can be made to fit any size of bedstead. The great feature of the invention is the jointed litter or stretcher. This is made in two parts or frames of equal size and covered with strong canvas; each part covers half the bed lengthways. To place the patient upon this stretcher two actions only are required. One part is slipped under his right side and the other under his left. The patient is then wound up to a convenient height by means of chair webbing suspended from the crossbar over the bed, at the end of which bar is a double threaded screw with capstan wheel, which is self fixing. The whole movement is simple, noiseless, and secure. Further, the patient can be raised, if desired, to any angle at either end by detaching the webbing at the opposite end. The advantage claimed for the invention is the absence of the numerous stretches of webbing commonly in use, which are placed separately under the patient before he can be raised at all, and which must more or less cause inconvenience, if not actual pain to the patient. Here, as described, only two slight movements are made, and the patient lies easily on the stretcher. The heavier the patient the tighter do the two halves of the stretcher lock from the action of the cross levers by which it is suspended. We saw the lift in action, and it seemed to us to be the best we have inspected. By it a heavy patient can be lifted off his bed, wheeled across the room and deposited on another bed or couch, or let down gently into a bath and without moving a limb. One nurse is quite capable of performing all these movements. We are quite sure that the inventors have only to show this lift in order to obtain a demand for it.

GOODMAN'S PATENT SURGICAL BANDAGES PIN.

This consists of a strip of nickled metal, at one end of which is the hinge of the double pin, at the other the catch. The metal is cut away at this latter end, so that when the pin is undermost its two sides can be compressed and pushed home in the catch. The advantages of this neat little adjunct to the surgeon's drawer are the security of a double pin, the impossibility of its coming unfastened, and the ease with which it can be made fast or loosened. Added to these is the plain fact that one is not likely to run the pin into one's fingers and thus set up a possible inoculation of septic matter. The pin is a decided addition to the nurse's chatelaine, and will, no doubt, be fully appreciated by all who have much bandaging and fastening off to do.

WODDERSPOON AND CO.'S TEMPERATURE AND DIET CHARTS.

We again have pleasure in drawing the attention of the profession to these most useful charts. We believe they were originally arranged by the late Dr. Gould and Mr. S. Benton. They are used in nearly all the chief hospitals, and their utility in fixing the hours of taking the temperature, feeding, giving medicine, noting any alteration in the patient's condition, &c., is so well known and appreciated that no words of ours are needed in their praise. We note a good four-hour chart among the specimens submitted to us. This in cases of extreme gravity is a valuable enlargement of the general twelve-hour plan. The charts are to be obtained at 7, Serle Street, Lincoln's Inn, and we may add that they seem to us to be very reasonable in price.

## Figures and Tables

**Figure f1:**